# Iron Content, Iron Speciation and Phycocyanin in Commercial Samples of *Arthrospira* spp.

**DOI:** 10.3390/ijms232213949

**Published:** 2022-11-12

**Authors:** Gloria Isani, Enea Ferlizza, Martina Bertocchi, Thomas Dalmonte, Simonetta Menotta, Giorgio Fedrizzi, Giulia Andreani

**Affiliations:** 1 Department of Veterinary Medical Sciences, Alma Mater Studiorum, University of Bologna, Via Tolara di sopra 50, 40064 Ozzano dell’Emilia, Italy; 2 Department of Experimental, Diagnostic and Specialty Medicine, Alma Mater Studiorum, University of Bologna, Via Belmeloro 8, 40126 Bologna, Italy; 3 Istituto Zooprofilattico Sperimentale della Lombardia e dell’Emilia Romagna, Chemical Department, Via P. Fiorini 5, 40127 Bologna, Italy

**Keywords:** *Arthrospira*, biochemical parameters, cyanobacteria, phycocyanin, trace elements

## Abstract

Cyanobacteria are characterized by high iron content. In this research, we collected ten commercial samples of *Arthrospira* spp. sold as food supplement to determine iron content and assess whether iron speciation showed variability among samples and changed respect to *A. platensis* grown in controlled conditions. Particular attention was also paid to phycocyanin, as an iron-binding protein. In six of the ten samples, 14 essential and non-essential trace elements were analysed using ICP-MS. Iron content measured in samples using atomic absorption spectrometry (AAS) varied from 353 (sample S5) to 1459 (sample S7) µg g^−^^1^ dry weight and was in the range of those reported by other authors in commercial supplements. Iron speciation was studied using size exclusion chromatography followed by the analysis of the collected fraction for the determination of iron by AAS and for protein separation using SDS-PAGE. Overlapping chromatographic profiles were obtained for total proteins, phycocyanin and iron, although quantitative differences were evidenced among the samples analysed. In most samples, iron was mainly bound to ligands with high molecular mass; however, in four samples iron was also bound to ligands with low molecular mass. In fractions containing the most relevant iron burden, the principal protein was phycocyanin, confirming its role as an iron-binding protein in commercial samples.

## 1. Introduction

*Arthrospira* spp. are photosynthetic cyanobacteria used as a dietary supplement (spirulina) due to their high content of high-quality proteins, including phycocyanin, pigments, essential trace elements, particularly iron, and other bioactive molecules [1]. This unique nutritional profile makes spirulina an attractive alternative and novel food [2].

Iron is an essential trace element for all living organisms. However, its redox activity poses significant challenges to cells: the reactivity of Fe^2+^ toward H_2_O_2_ determines the formation of the hydroxyl radical (HO•). For this reason, in biological systems iron is not found in the state of free ion but always bound to proteins involved in sophisticated homeostatic control mechanisms [3]. Iron is of special relevance for photosynthetic cyanobacteria, which have evolved complex uptake mechanisms [4] to cope with the low concentrations of this essential metal in their environments and are able to bioaccumulate iron concentrations of four to six orders of magnitude higher than those of the aquatic environment in which they grow [5]. Bacteria utilizes two types of ferritin-like molecules to store iron in the trivalent state as ferric ions, namely bacterial ferritin (Ftn) and bacterioferritin (Bfr) [6]. Alternatively, iron can be stored as inorganic iron. It has been reported that *Arthrospira platensis* trichomes accumulate iron mostly in the form of Fe^3+^ as ferrihydrite [7]. In cyanobacteria, due to these different storage possibilities, iron can be stored in high concentrations, at the same time protecting cell from potentially harmful Fe^2+^/Fe^3+^ redox cycling. 

Different growing conditions and environmental parameters dramatically impact the biochemical composition of the biomass, determining wide variations among commercial products based on *Arthrospira* [8,9]. Therefore, in addition to research performed in controlled laboratory conditions or pilot studies, there is a need for analyses and comparisons among commercial samples of different origins and manufacturers. Moreover, commercial products based on *Arthrospira* are subjected to processing and packaging which can modify the biochemical composition, further affecting the nutritional quality. In previous research using *A. platensis* F&M-C256 strain reared in optimal and controlled conditions, we focused on the effects of varying iron concentrations in the culture medium on iron speciation and iron-binding proteins [10]. In this research, we collected ten commercial samples of *Arthrospira* spp. sold as food supplements to determine iron content and to assess whether iron speciation showed variability among samples or changed respect to *A. platensis* F&M-C256, using different molecular approaches. Particular attention was paid to phycocyanin, the most abundant protein of *Arthrospira*, as a candidate iron-binding protein. Finally, 14 essential and non-essential trace elements were measured in samples from Italy and China.

## 2. Results and Discussion

### 2.1. Iron Content in Commercial Samples

Iron content is reported in Table 1. The obtained values varied between 353 (sample S5) and 1459 µg g^−^^1^ dry weight (dw) (sample S7), with a mean value (±SD) of 608 (±611). The high standard deviation among samples is due to the high iron content detected in sample S7 from China, which has an iron content two to four times higher than the other samples. A similar outcome was reported by Kejžar et al. (2021) [9] for a spirulina-based supplement from Hawaii, which had an iron content as high as 3.29 ± 0.27 mg g^−^^1^dw. Nevertheless, the iron content falls within the range of those reported by other authors. Principe et al. (2020) [8] reported values from 63 to 1066 µg g^−^^1^ dw in seven dietary supplements sold in Argentina. Kejžar et al. (2021) [9] found a mean iron content of 1360 ± 1330 µg g^−^^1^ in dietary supplements sold in Slovenia. In 46 spirulina supplements available on the Slovenian market, an iron content from 370 to 3480 µg g^−^^1^ dw was reported by Rutar et al. (2022) [11]. These data highlight the high variability of iron in supplements based on *Arthrospira* spp. and *Spirulina* spp. It is well known that the iron concentrations in the medium used for the cultivation of cyanobacteria is able to determine wide variations of the metal content in the biomass [10,12].

### 2.2. Protein Fractionation and Iron Speciation

The ten samples were extracted to analyse the distribution of iron between the soluble and insoluble fractions. The percentages are reported in Table 1. In all the samples, iron was more present in the pellet than in the soluble fraction. The percentage of iron in the soluble fraction varied from 30% in sample S1 to 7% in sample S7, with most samples presenting a percentage between 14 and 18%. Low percentages of iron from 6 to 10% were also detected in soluble fractions of extracts from *A. platensis* F&M-C256 strain reared in controlled laboratory conditions [10]. Taken together, these data argue in favour of the suggestion reported by Perfiliev et al. (2018) [7] that *A. platensis* trichomes accumulate iron mostly in an inorganic form as ferrihydrite. This compound could be responsible of the high iron percentages determined in the pellet fraction of all samples examined.

**Figure 1:**
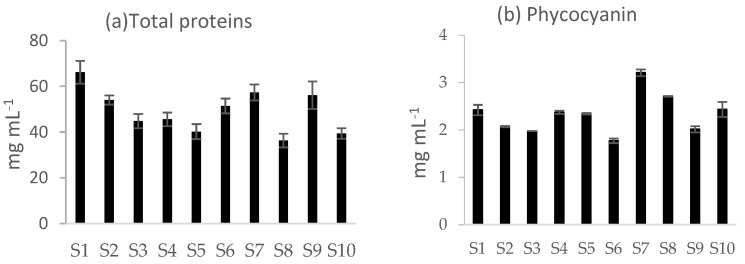
Concentration of total proteins (**a**) and phycocyanin (**b**) in *Arthrospira* spp. soluble fraction of commercial samples. Data are expressed in mg mL^−^^1^ and are reported as mean ± SD (n = 3).

In addition to inorganic compounds, iron can bind to biomolecules, of which proteins, amino acids, and heme are of particular interest. All these molecules are present mostly in the soluble fraction of extracts. The concentration of total proteins varied from 36.3 mg mL^−^^1^ in sample S8 to 66.2 mg mL^−^^1^ in sample S1 (Figure 1a), while the concentration of phycocyanin varied from 1.77 mg mL^−^^1^ in sample S6 to 3.21 mg mL^−^^1^ in sample S7 (Figure 1b).

**Figure 2:**
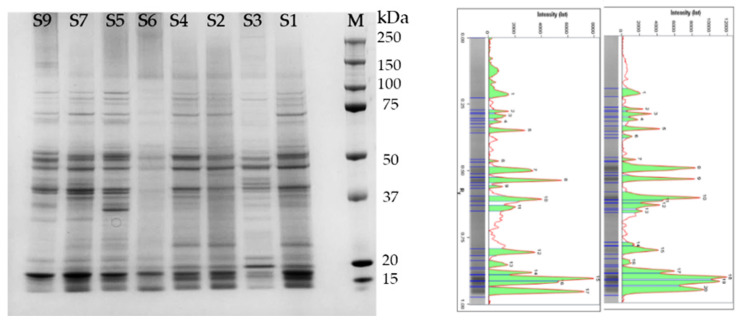
The 1D proteomics analysis of *Arthrospira* spp. soluble fractions from commercial samples. Proteins (3 µg) were separated using SDS-PAGE (12%) and stained with Coomassie^®^ Brilliant Blue R-250. Left: representative gel; lanes 1–8: soluble extracts of samples S1, S2, S3, S4, S5, S6, S7 and S9; lane 10 (M): molecular mass marker (Precision Plus Protein Standard, Biorad); right: representative pherograms obtained from samples S4 and S2.

To obtain a general insight on the quality of the extracts, total proteins present in soluble fraction were separated using SDS-PAGE (Figure 2). Despite the different origin and formulation of the samples, the profiles obtained were similar and indicative of a good quality, without sign of protein degradation, despite the storage of commercial samples at room temperature. The quality of these extracts was higher than that reported by Jin et al. (2020) [13] for spirulina powder extracts. Those authors obtained only three diffuse bands at 42, 37, and <25 kDa after SDS-PAGE, while in the samples analysed in the present research different common bands ranging from 250 to <15 kDa were evidenced, comparable to those present in extracts from *A. platensis* F&M-C256 strain reared in controlled laboratory conditions [10]. Most of the protein bands were in the ranges between 35–50 and 15–20 kDa; similar ranges were reported by Ismaiel et al. (2018) [14].The most abundant band at 18 kDa is the subunit of phycocyanin. The intensity of the band shows wide variations among the samples, from samples S1 and S7, which present the most intense bands, to sample S3, which shows only a faint band. On the other hand, this sample (S3) presents a band at 20 kDa, more evident than that of all the other samples. The electrophoretic profiles can be considered an interesting molecular fingerprint to evaluate the quality and integrity of proteins in supplements based on *Arthrospira* spp. and its use should be encouraged.

**Figure 3:**
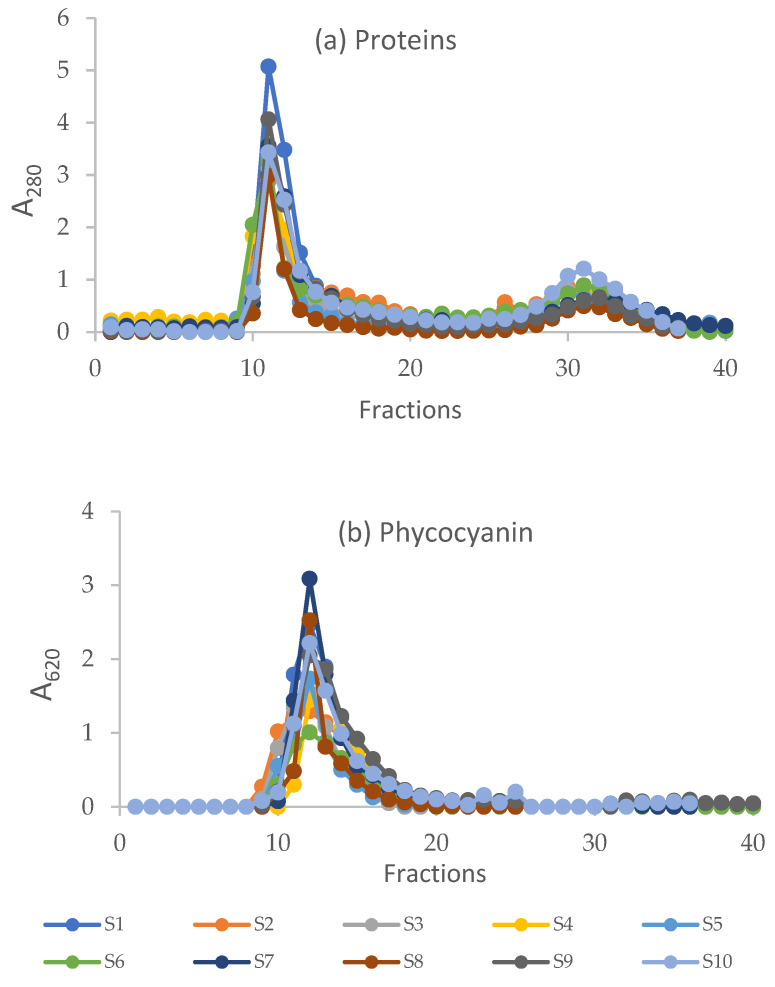
Chromatographic pattern after the size exclusion chromatography of extracts obtained from samples S1–S10. Total proteins were detected at 280 nm (**a**); phycocyanin was detected at 620 nm (**b**).

The study of iron speciation was addressed by combining a chromatographic fractionation of proteins using size-exclusion chromatography (SEC) associated with sensitive metal detection in the fractions using AAS, a hyphenated approach which is commonly used in metallomics studies [15]. Total proteins and phycocyanin in fractions obtained after SEC are reported in Figure 3. Overlapping chromatographic profiles were obtained in all the samples analysed, although differences were evidenced. 

Regarding proteins, the first peak is present between fractions 10 and 15, with maximum absorbance at 280 nm measured in fraction 11, correlated with the concentration of total proteins measured in the soluble extracts (Figure 1) (*p* < 0.05). This peak contains proteins with high molecular mass (MM) between >75 to 40 kDa. A second peak of absorbance is present in fractions 29–34. In these fractions, small peptides and free amino acids elute, including the so-called mycosporine-like amino acids (MAAs). These secondary metabolites are low molecular mass (<400 Da) molecules produced by a wide range of organisms and are characterized by strong UV absorption maxima between 310 and 362 nm. Mycosporine-like amino acids have the function of absorbing ultraviolet radiation, protecting organisms from the possible damage caused by exposure to the sun [16]. The presence of these amino acids in the peak was verified by measuring the absorbance at different wavelengths between 310 and 360 nm. The data are reported in Supplementary Figure S1. The chromatographic profiles obtained at 280 nm are comparable to those reported for *A. platensis* F&M-C256 grown in optimal laboratory conditions [10].

**Figure 4:**
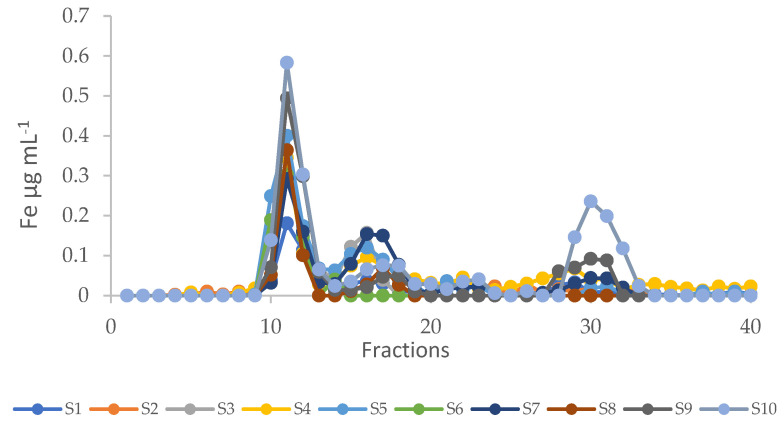
Iron chromatographic profiles after size exclusion chromatography of extracts from samples S1–S10. Iron concentration is expressed as µg mL^−^^1^.

Iron profiles after SEC are reported in Figure 4. In all the samples, two main peaks were present, namely in fractions 10–13 and in fractions 15–18, except for sample S6, which presented only the first peak. Iron distributed differently between these two peaks, depending on the sample analysed. In most samples, iron was mainly bound to ligands with high molecular mass (HMM) between >75 and 60 kDa (fractions 10–12). Differently, in samples S3 and S7, a consistent percentage of the metal (34% and 40%, respectively) was bound to ligands with intermediate molecular mass (IMM) between 40 and 20 kDa (fractions 15–18). In samples S4, S7, S9, and S10, iron was also bound to ligands with low molecular mass (LMM) (fractions 28–32). Particularly, in sample S10, a consistent percentage of iron (32%) was detected in these last fractions. This peak was overlapping with the peak of MAAs (Figure 2a and Figure S1). Recently, Varnali et al. (2022) [17] reported the computational analysis of stable iron-complexed MAA structures, supporting previous suggestions made in the literature on the potential iron-chelating ability of MAAs. The iron peak present in fractions 28–32 could be considered the first evidence of the iron-binding capacity of MAAs in *Arthrospira*. However, the presence in these fractions of other LMM iron-binding molecules, as other free amino acids, cannot be excluded.

Contrasting results have been reported on the molecular ligands of iron in cyanobacteria. Cepoi et al. (2018) [18] reported that in *A. platensis* grown in a medium containing Fe_3_O-glycin (50 mg L^−^^1^), high percentages of iron were bound to organic ligands: 43% was bound to proteins and 24% to free amino acids. The presence of an iron peak in the fractions of LMM ligands found in this study could be indicative of iron bound to amino acids or small peptides, as discussed above. On the contrary, Perfiliev et al. [7] reported that *A. platensis* accumulates iron mainly as Fe^3+^ in an inorganic form, such as ferrihydrite. Accordingly, Rutar et al. (2022) [11] showed that most iron (82–92%) found in spirulina dietary supplements was present as Fe^3+^. The authors concluded that the bioavailability of iron from the supplements is low, as only a small amount of the metal is present as a more bioavailable ferrous ion, Fe^2+^. The partitioning of iron between the pellet and the soluble fraction found in most of the samples analysed in this study, except for sample S1, showed that percentages of the metal between 78 and 93% are present in the pellet, presumably as inorganic iron. These data are in accordance with those reported by Rutar et al. (2022) [11]. However, different suggestions were made by Puyfoulhoux et al. (2001) [19], who compared the iron bioavailability of iron-fortified spirulina to different iron sources. Those authors measured ferritin formation in Caco-2 cells exposed to digests obtained from meat, yeast, spirulina, and wheat. The results obtained were indicative of a metal bioavailability for spirulina not significantly different from that exhibited by the meat digest, despite the different chemical form of iron.

### 2.3. SDS-PAGE of Fractions after SEC

**Figure 5:**
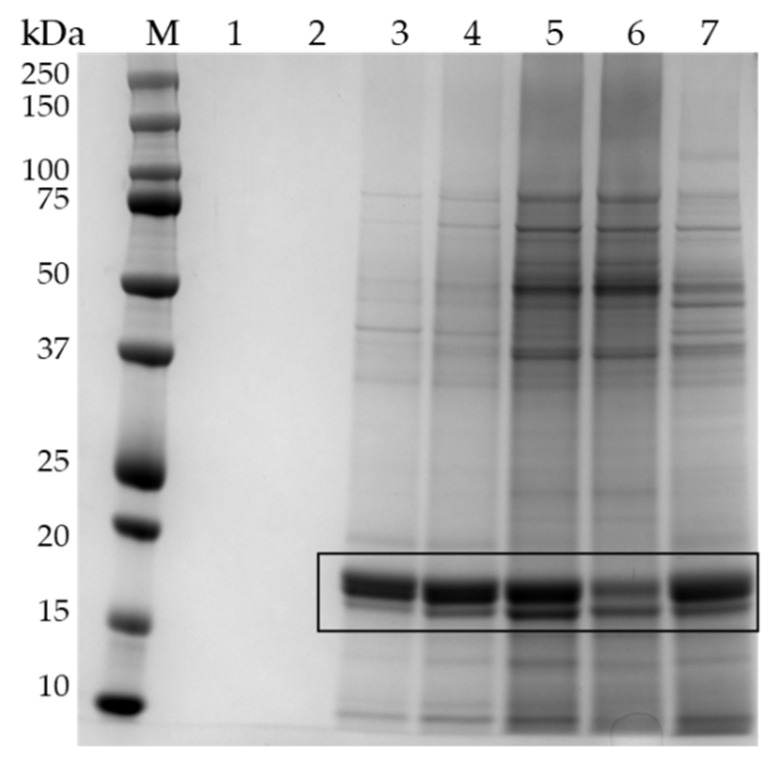
Representative SDS-PAGE gel (4–12%, Coomassie staining) of proteins contained in fractions obtained after size exclusion chromatography of cellular extracts. M: molecular mass marker; lanes 1–2: fraction 31 of samples S9 and S10; lanes 3–7: fraction 11 of samples S8, S4, S1, S9, and S7, respectively. Rectangle indicates the bands at 18 and 17 kDa containing the subunits of the phycocyanin.

To shed more light on iron-binding proteins, fraction 11, which contains the most relevant metal burden, underwent SDS-PAGE on 4–12% gels. A representative gel is reported in Figure 5. Fraction 11, which had the highest absorbance at 280 nm and the highest iron concentration, showed different protein bands, with an apparent molecular mass ranging from 75 to 10 kDa. The most represented bands are at 18 and 17 kDa (Figure 5, lanes 3–7). These bands contain C-phycocyanin (C-PC) [10]. This multimeric blue protein belonging to the phycobiliprotein family presents two subunits, which bind a particular prosthetic group, phycocyanobilin, similar to bilverdin and bilirubin. In addition to functioning as a light-capturing antenna for photosystem II, this protein is able to bind iron in vitro [20] and in vivo [10]. The data of our study indicate that iron is bound to C-PC in commercial products sold as nutritional supplements, despite harvesting, processing, and packaging process that could modify iron speciation and the biochemical profile of cyanobacteria. 

Fraction 31 from samples S9 and S10, despite a significant iron burden, did not contain proteins (Figure 5 lanes 1–2); other small molecules are responsible for iron binding in this fraction (Figure 4), presumably amino acids, as discussed above.

### 2.4. Essential and Non-Essential Trace Elements Content

Commercial supplements based on *Arthospira* are interesting sources of macro and trace elements. However, many producers are located in Asia, particularly in India and China, and use cultivation practices not subjected to strict regulation. High contents of aluminium, lead, and inorganic arsenic due to environmental contamination or harvesting systems have been reported [21,22]. For this reason, we decided to compare three samples of *Arthospira* spp. grown in Italy (S3, S5, and S6) with three samples from China (S1, S4, and S7).

The mean content of essential trace elements presented significant differences between samples from Italy and China (Table 2). This decreasing order was present in Italian samples: Fe > Zn > Cu > Mn > Ni > Cr > Mo > Co > Se, while in Chinese samples elements followed this order: Fe > Mn > Zn > Ni > Cu > Cr > Co > Se > Mo and were characterized by a significantly higher content of manganese and a significantly lower content of copper and cobalt (Table 2). High levels of manganese were previously reported by Rzymski et al. (2019) [22] and Rutar et al. (2022) [11], while Sandgruber et al. (2021) [23] found in some commercial samples of *A. platensis* a manganese content < LOQ. The ability of cyanobacteria to bioaccumulate metals is well known and the variability is strictly related to the metal concentration and bioavailability in the water. Ghanbarzadeh et al. (2022) [24] reported that *A. platensis* MGH-1 is able to accumulate manganese levels as high as 3 g kg^−1^ when exposed to a manganese concentration of 40 mg L^−1^ in the culture medium.

Some peculiarities emerge from the analysis of other trace elements. For example, a mean selenium content of 0.34 µg g^−1^ dw was detected in samples from China, with sample S7 with a content as high as 0.71 µg g^−1^dw. These values are in the range of those reported for grains and cereals [25] and spirulina could be considered an interesting integrative source of selenium for vegan individuals. However, besides the total intake of dietary selenium, the element speciation may also be important and requires future studies. 

Regarding non-essential trace elements, aluminium content with a mean value of 104.7 µg g^−1^ was higher in samples from China compared to samples from Italy. This difference could be related to cultivation and harvesting systems. Adding chemicals to induce flocculation is a method widely used to harvest microalgal biomass [26] and aluminium chloride salts are the most efficient for this purpose [27]. The aluminium content found in two samples from China is of particular concern because this metal has been recently classified by IARC as a carcinogen for humans. The discussion of possible health risks posed by the consumption of these supplements is out of the scope of the present research. However, to avoid biomass contamination, alternative physical or biological harvesting methods are suggested.

The content of cadmium and lead in samples was low, below the maximum level (1.0 mg kg^−1^) set by the European Commission [28], and indicative of low environmental contamination. The data obtained in the supplements analysed in this research are in accordance with those recently reported by other authors [22,29].

## 3. Materials and Methods

### 3.1. Commercial Samples of Arthrospira and Sample Preparation

Commercial samples from different companies were purchased in Italy. A total of ten samples (S1–S10) were analysed (Table 3). The tablets were ground using a mortar and pestle. Meanwhile, the capsules were opened and powders were directly used for analysis.

### 3.2. Iron Analysis

To avoid contamination, all the reagents were handled carefully; polyethylene disposables were thoroughly washed with HCl 1 N under a fume hood. All the reagents were from Merck (Darmstadt, Germany); the acids were of Suprapur grade. Commercial samples (400 mg) and pellets (200 mg) obtained as described in Section 3.3 were placed in individual acid-washed Teflon jars and were digested with the protocol optimized for metals [30]. Briefly, samples were added with 1–2 mL 65% HNO_3_ and 0.25–0.5 mL 30% H_2_O_2_, digested in a microwave oven, transferred into 5–10 mL polyethylene volumetric flasks, and analysed using a flame atomic spectrophotometer equipped with a deuterium lamp background correction (AAnalyst 100, Perkin Elmer, Waltham, MA, USA). Supernatants obtained as described in Section 3.3 were diluted 1:10 with MilliQ water and directly analysed without any further treatment. The accuracy of the method was evaluated with ERM^®^–BB422 fish muscle. The concentrations found with the method used in this study fell into the certified uncertainty interval given by ERM, corresponding to a 95% confidence level. The iron detection limit was 0.04 μg mL^−1^. Iron concentrations were reported as μg mL^−1^ or μg g^−1^ dw depending on the sample analysed.

### 3.3. Iron Speciation and Size Exclusion Chromatography (SEC)

One hundred milligrams of commercial samples were crushed in a mortar with pestle under liquid nitrogen and homogenized in 30 volumes (*w*/*v*) of Tris–HCl 20 mM and 10 mM mercaptoethanol, pH 8.6, using an Ultraturrax (IKA, Staufen, Germany) homogenizer. The homogenate was sonicated for 10 min at 38 kHz and then centrifuged at 20,000× *g* for 40 min at 4 °C, obtaining the separation between supernatant (soluble fraction) and pellet. For each extract, a volume of 0.8 mL supernatant was applied to a Sephadex G-75 chromatography column (0.9 × 90 cm). The column was calibrated using a commercial kit (GF70-1KT, Sigma-Aldrich, St Louis, MO, USA). Fractions of 1.5 mL were collected and analysed for iron concentration using direct aspiration of the solution into the flame of an atomic absorption spectrophotometer, as described above (Section 3.2). The pellets obtained from the centrifugation of the homogenate were digested in a microwave oven, as reported above (Section 3.2), and iron concentration was determined using a flame atomic absorption spectrophotometer (AAnalyst 100, Perkin Elmer, Waltham, MA, USA) as reported in Section 3.2, “Iron analysis”.

The concentration of total proteins in soluble fractions was determined using Lowry assay (DC Protein Assay, Biorad, Hercules, CA, USA) following the manufacturer’s instruction, while phycocyanin concentration was calculated after reading the absorbance at 615 and 652 nm using the following formula [31]:

(A_615_ − 0.474 × A_652_)/5.34

In fractions obtained from size exclusion chromatography, total proteins, phycocyanin, and mycosporine-like amino acids were determined by direct measurement of the absorbance at 280, 615, and 310–360 nm, respectively (DeNovix DS-11 Series Spectrophotometer, Wilmington, DE, USA).

### 3.4. SDS-PAGE

Three µg of proteins were loaded onto 4–12% Bis-Tris polyacrylamide gels (NuPage/Thermo Fisher Scientific, Waltham, MA, USA), and electrophoresis (PAGE) was carried out in an Xcell SureLock Mini-Cell with 2-(N-morpholino) ethanesulfonic acid buffer (MES; NuPage/Thermo Fisher Scientific, Waltham, MA, USA), containing sodium dodecyl sulphate (SDS). Each gel was also loaded with standard proteins of known molecular weight (Precision Plus Protein^TM^ Dual Color Standard, Bio-Rad, Hercules, CA, USA). The electrophoresis was performed following the procedure reported by Isani et al. (2022) [10]. 

### 3.5. Trace Elements Analysis

The samples were digested using a wet procedure in polypropylene tubes (Digi TUBES SCP Science, QC, Canada). Ten mL of 70% nitric acid (J.T. Baker Instra-Analyzed™) were added, and the tubes were placed in Digi-Prep graphite Digestion Blocks (SCP Science, Baie-D’Urfé, QC, Canada) at 75 ± 5 °C overnight. After cooling, the clear solutions were diluted to 20 mL with high purity deionized water (Evoqua Water Technologies, Günzburg, Germany). The samples were additionally diluted with a solution of 2% nitric acid and 0.5% hydrochloric acid (Sigma, Suprapur, St Louis, MO, USA). The analyses were carried out using inductively coupled plasma mass spectrometry (ICP-MS 7700 Series Agilent Technologies Inc., Santa Clara, CA, USA) with an ASX-500 CETAC Autosampler (Cetac Technologies, Omaha, NE, USA) following the procedure reported by Andreani et al. (2019) [32].

The accuracy of the method was determined by analysing certified reference material (Joint Research Centre BCR-185R Bovine Liver) in each batch. The concentration values of the reference materials fell within the confidence interval given by the Joint Research Centre (Brussels). For each series of analyses, a white sample (acid used for sample mineralization) was mineralized and treated as described above; the limit of quantification (LOQ) was 0.005 µg/g and the limit of detection (LOD) was 0.003 µg/g for each analysed element. The results were expressed in µg/g dry weight (dw).

### 3.6. Statistical Analysis

Statistical analysis was carried out using statistical software (RStudio-1.2.1335 Statistical and R, R version 4.2.1, Vienna, Austria). All data were evaluated using standard descriptive statistics and are reported as mean ± standard deviation (SD). The comparison between Italian and Chinese samples was performed with *t*-test, and a *p*-value < 0.05 was considered significant.

## 4. Conclusions

The results obtained indicate that the commercial samples maintained a good biochemical profile, despite harvesting, processing, packaging processes, and storage at room temperature. The electrophoretic pattern of proteins from soluble extracts and the chromatographic profiles are comparable to those obtained from biomass grown in optimal laboratory conditions. The molecular approach based on different and hyphenated techniques allowed the production of a comprehensive fingerprint useful to compare the supplements analysed.

The high variability of iron in supplements based on *Arthrospira* spp. is confirmatory of data previously reported by other authors. The nature of iron-binding ligands and the bioaccessibility of the metal is still an open question, and further research is needed to address this important issue and shed more light on the complex relation between iron and iron-binding molecules in cyanobacteria. Of particular interest is the confirmation that phycocyanin maintain the ability to bind iron also in commercial products, opening new perspectives to the use of this protein in supplements. However, other low molecular weight ligands, such as mycosporine-like amino acids, could be involved in iron-binding and should be considered in future studies.

## Figures and Tables

**Table 1:** Iron content in ten commercial samples (S1–S10) of *Arthrospira* spp. sold as dietary supplement. Data are expressed as µg g^−1^ dw and are reported as mean ± SD (n = 3). The distribution of iron between the pellet and the soluble fraction (sf) is reported as percentage.

	[Fe]	Fe	Fe
	(µg g^−1^ dw)	% pellet	% sf
S1	467 ± 15	70	30
S2	782 ± 27	84	16
S3	440 ± 30	84	16
S4	540 ± 22	83	17
S5	353 ± 18	86	14
S6	474 ± 2	86	14
S7	1459 ± 17	93	7
S8	538 ± 19	82	18
S9	387 ± 20	78	22
S10	461 ± 16	82	18

**Table 2:** Trace element content in six selected commercial samples. Samples S1, S4, and S7 were produced in China (CN), while samples S3, S5, and S6 were produced Italy (IT). Data are expressed as µg g^−1^dw. The *p*-value for comparison between Italian and Chinese samples is reported; a *p* -value < 0.05 was considered significant.

Sample	Fe	Zn	Cu	Mn	Ni	Cr	Mo	Co	Se	Al	Pb	As	Cd	Hg
S3 (IT)	432	65.3	10.3	10.1	8.17	0.99	0.09	0.06	0.04	3.83	0.13	0.12	0.01	0.01
S5 (IT)	367	28.9	7.23	7.19	6.06	0.24	0.09	0.06	nd	1.46	0.04	0.01	0.01	nd
S6 (IT)	448	34.3	9.92	7.27	2.52	0.94	0.09	0.07	0.02	8.94	0.17	0.08	0.02	nd
mean	416	42.8	9.16	8.22	5.58	0.72	0.09	0.06	0.03	4.74	0.11	0.07	0.02	-
SD	43.1	19.7	1.68	1.70	2.86	0.42	0.00	0.01	0.01	3.82	0.07	0.06	0.00	-
S1 (CN)	517	16.8	0.65	21.7	0.61	0.21	0.10	0.20	0.10	21.2	0.13	0.46	0.01	nd
S4 (CN)	576	10.5	2.13	34.7	5.83	0.41	0.11	0.45	0.14	176	0.47	1.46	0.11	0.02
S7 (CN)	1518	11.4	1.89	36.8	0.68	1.36	0.14	0.38	0.71	117	0.55	0.36	0.02	0.01
mean	870	12.9	1.56	31.1	2.37	0.66	0.11	0.34	0.32	105	0.38	0.76	0.05	0.01
SD	561	3.41	0.79	8.20	2.99	0.61	0.02	0.13	0.34	77.9	0.22	0.61	0.06	0.01
*p*-value	0.234	0.060	*0.002*	*0.009*	0.250	0.892	0.133	*0.020*	0.336	0.091	0.115	0.122	0.384	-

nd: not detected.

**Table 3:** Origin and form of analysed samples.

Sample	Species	Origin	Form	
S1	*Arthrospira maxima*	China	powder	Non-organic
S2	*Arthrospira maxima*	Italy	tablet	Non-organic
S3	*Arthrospira platensis*	Italy	powder	Non-organic
S4	*Arthrospira maxima*	China	powder	Non-organic
S5	*Arthrospira platensis*	Italy	pellet	Non-organic
S6	*Arthrospira platensis*	Italy	powder	Non-organic
S7	*Arthrospira platensis*	China	powder	Organic
S8	*Arthrospira platensis*	Italy	powder	Organic
S9	*Arthrospira platensis*	Italy	powder	Non-organic
S10	*Arthrospira platensis*	India	capsule	Organic

## Data Availability

Not applicable.

## Supplementary Materials

The following supporting information can be downloaded at: https://www.mdpi.com/article/10.3390/ijms232213949/s1.
