# Supplementary material for: Iron Content, Iron Speciation and Phycocyanin in Commercial Samples of Arthrospira spp
Source: Int J Mol Sci. 2022 Nov 12;23(22):13949. doi: 10.3390/ijms232213949 (PMC9698952; doi:10.3390/ijms232213949)
Supplement: Supplementary file 1 [file ijms-23-13949-s001.zip › Supplementary Figure S1.pdf]

Supplementary Figure S1: Chromatographic pattern of mycosporine-like aminoacids after size exclusion chromatography of extracts obtained from samples S1-S10.

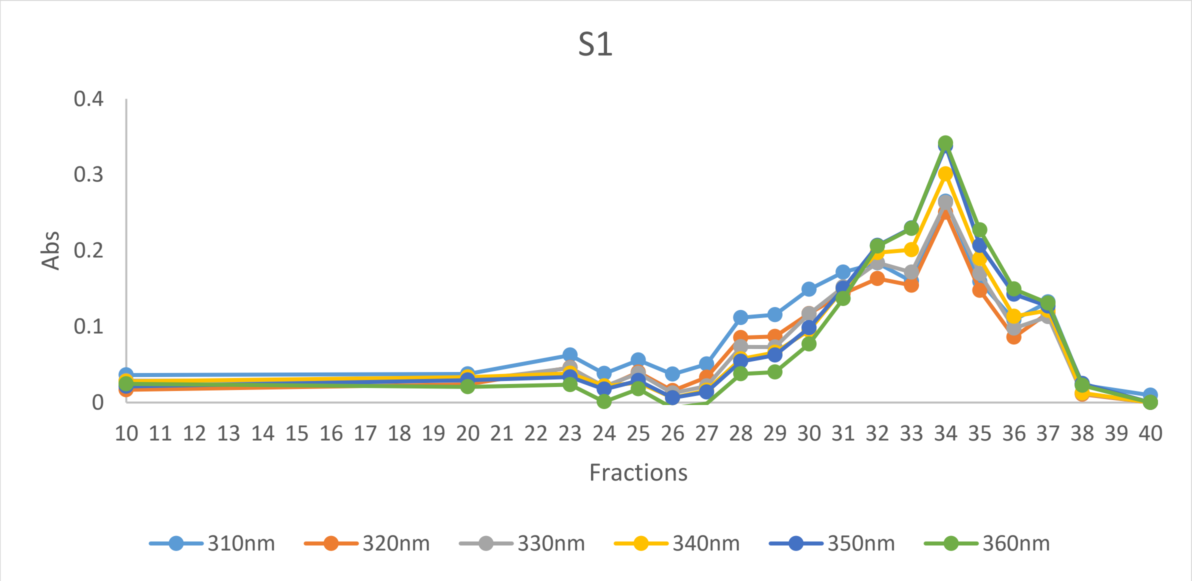

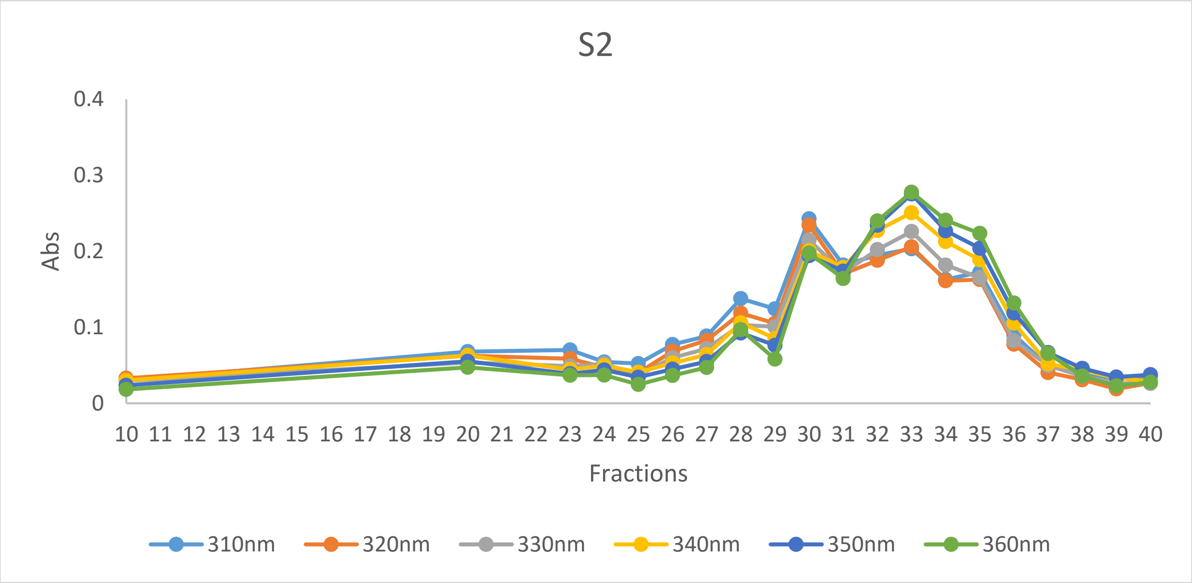

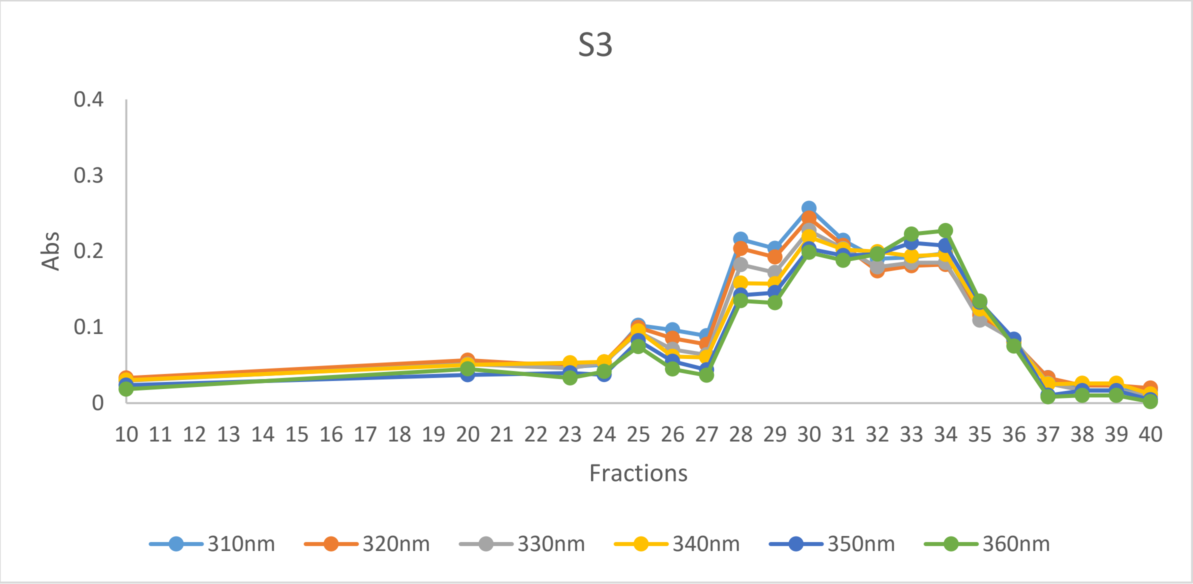
S3
Abs
0.4
0.3
0.2
0.1
0
10 11 12 13 14 15 16 17 18 19 20 21 22 23 24 25 26 27 28 29 30 31 32 33 34 35 36 37 38 39 40
Fractions
310nm
320nm
330nm
340nm
350nm
360nm

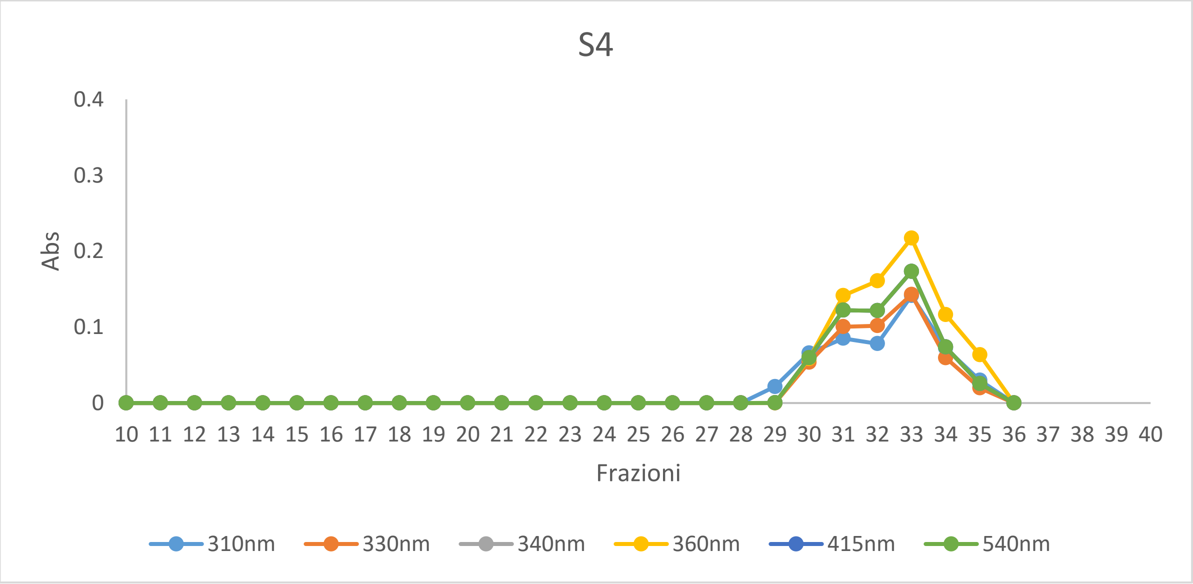
S4
Abs
0.4
0.3
0.2
0.1
0
10 11 12 13 14 15 16 17 18 19 20 21 22 23 24 25 26 27 28 29 30 31 32 33 34 35 36 37 38 39 40
Frazioni
310nm
330nm
340nm
360nm
415nm
540nm

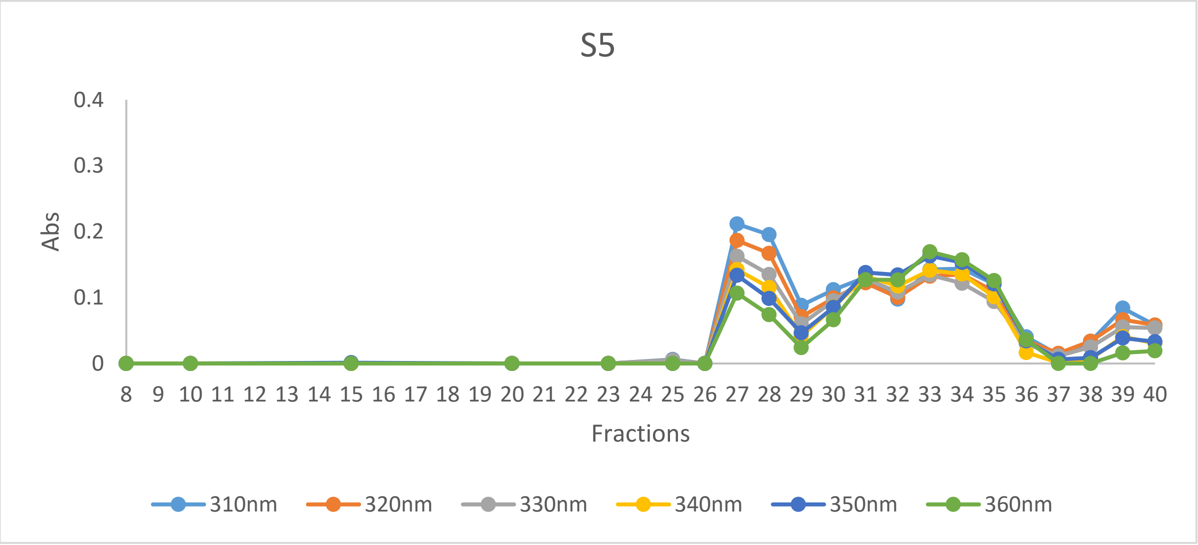
S5
Abs
0.4
0.3
0.2
0.1
0
8 9 10 11 12 13 14 15 16 17 18 19 20 21 22 23 24 25 26 27 28 29 30 31 32 33 34 35 36 37 38 39 40
Fractions
310nm
320nm
330nm
340nm
350nm
360nm

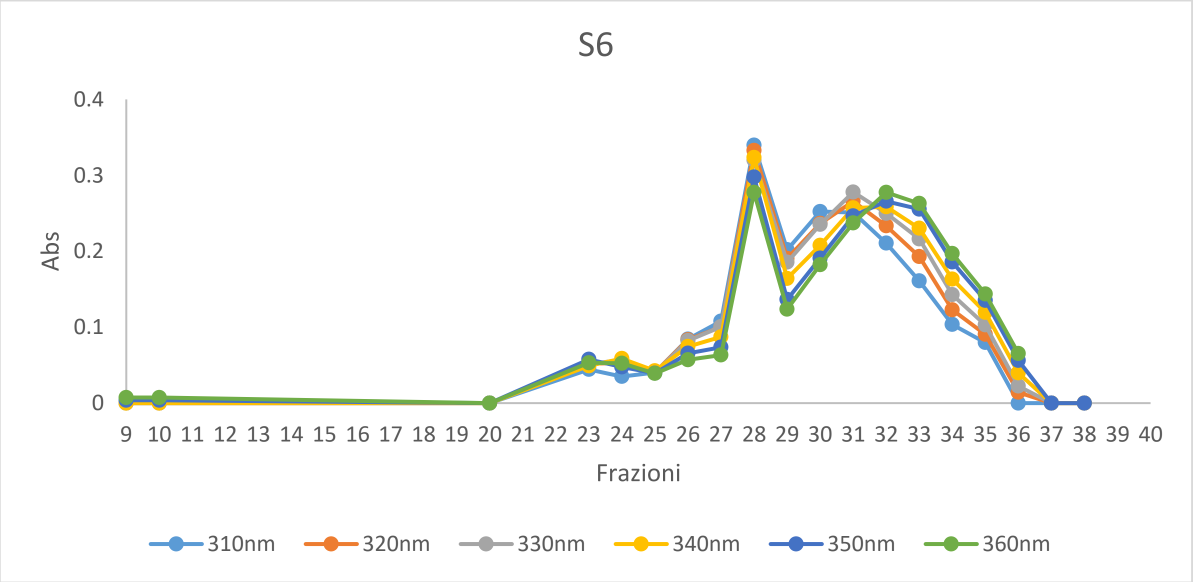
S6
0.4
0.3
0.2
0.1
0
Abs
9 10 11 12 13 14 15 16 17 18 19 20 21 22 23 24 25 26 27 28 29 30 31 32 33 34 35 36 37 38 39 40
Frazioni
310nm
320nm
330nm
340nm
350nm
360nm

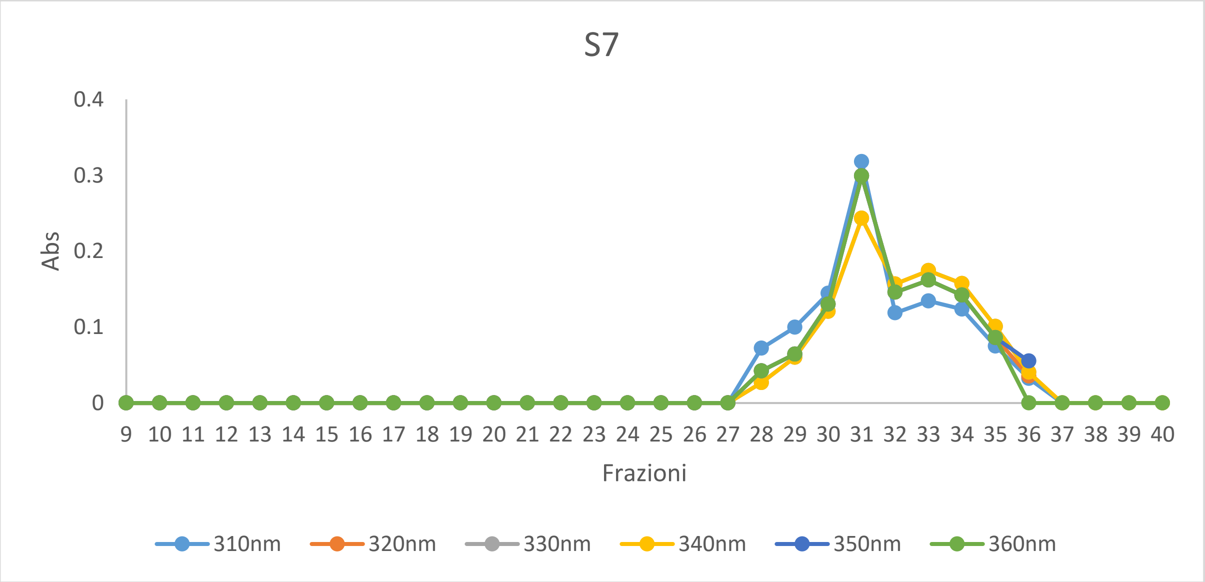
S7
0.4
0.3
0.2
0.1
0
Abs
9 10 11 12 13 14 15 16 17 18 19 20 21 22 23 24 25 26 27 28 29 30 31 32 33 34 35 36 37 38 39 40
Frazioni
310nm
320nm
330nm
340nm
350nm
360nm

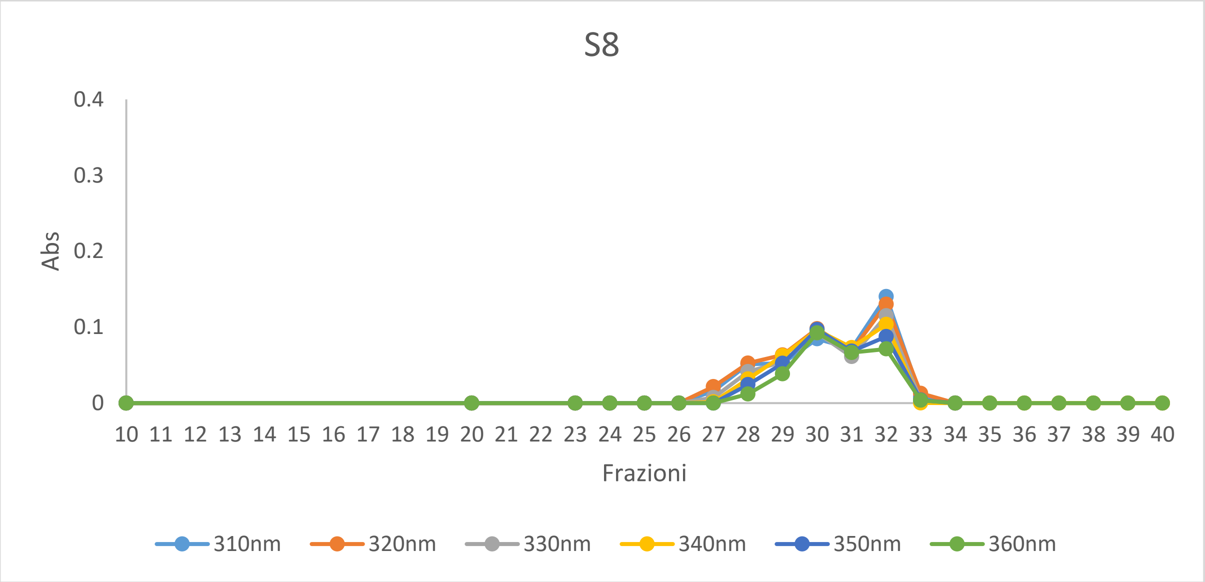
S8
Abs
0.4
0.3
0.2
0.1
0
10 11 12 13 14 15 16 17 18 19 20 21 22 23 24 25 26 27 28 29 30 31 32 33 34 35 36 37 38 39 40
Frazioni
310nm
320nm
330nm
340nm
350nm
360nm

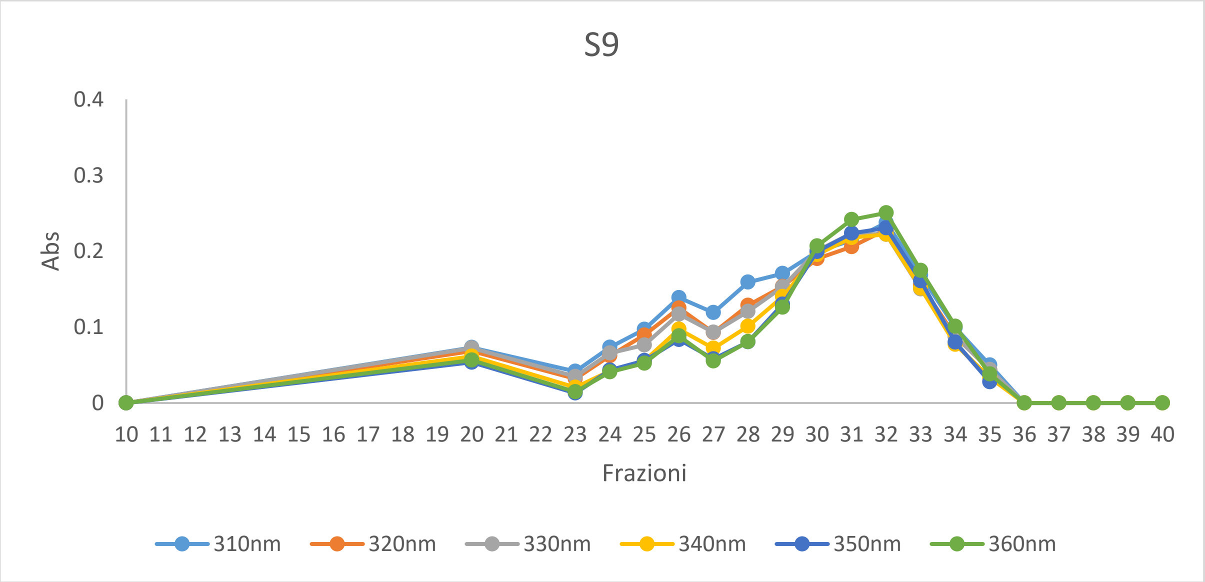
S9
Abs
0.4
0.3
0.2
0.1
0
10 11 12 13 14 15 16 17 18 19 20 21 22 23 24 25 26 27 28 29 30 31 32 33 34 35 36 37 38 39 40
Frazioni
310nm
320nm
330nm
340nm
350nm
360nm

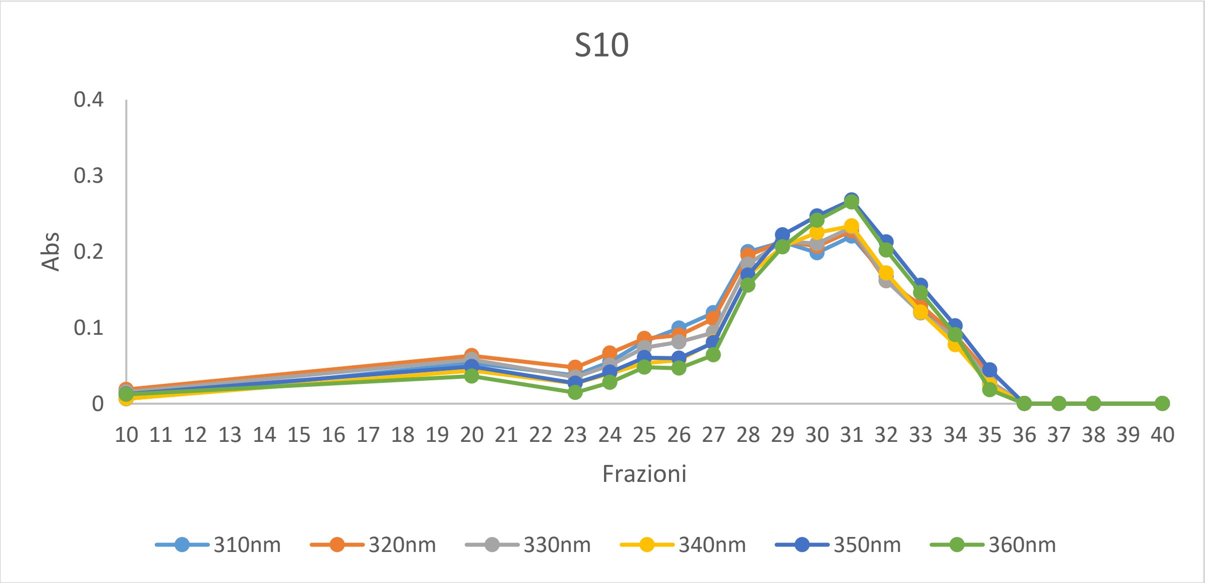
S10
Abs
0.4
0.3
0.2
0.1
0
10 11 12 13 14 15 16 17 18 19 20 21 22 23 24 25 26 27 28 29 30 31 32 33 34 35 36 37 38 39 40
Frazioni
310nm
320nm
330nm
340nm
350nm
360nm
